# Abiotic Methane Production Driven by Soil Reactive Oxygen Species

**DOI:** 10.1021/acs.est.6c01306

**Published:** 2026-04-06

**Authors:** Zi-Yan Liu, Hao Liu, Shi-Yu Zhang, You Feng, Ling-Li Wei, Dongmei Zhou, Zheng Chen

**Affiliations:** † Department of Health and Environmental Sciences, 122238 Xi’an Jiaotong-Liverpool University, 111 Ren’ai Road, Suzhou, Jiangsu 215123, P. R. China; ‡ Department of Chemistry, University of Liverpool, Crown Street, Liverpool L697ZD, United Kingdom; § Centre for Metabolomics Research, Department of Biochemistry, Cell and Systems Biology, Institute of Systems, Molecular and Integrative Biology, University of Liverpool, Liverpool L697BE, United Kingdom; ∥ State Key Laboratory of Pollution Control and Resource Reuse, School of the Environment, 12581 Nanjing University, Nanjing, Jiangsu Province 210023, P. R. China

**Keywords:** abiotic methane production, reactive oxygen species, carbon transformation, wetland soils, redox fluctuation, greenhouse gas

## Abstract

Methane (CH_4_), which is a potent greenhouse gas, is predominantly produced in wetland soils through biological processes. Recent studies reveal that reactive oxygen species (ROS) can abiotically generate CH_4_ via oxidative demethylation of organic compounds; yet, the environmental significance of this pathway remains unexplored. Here, we investigate the potential for reactive oxygen species (ROS)-driven CH_4_ formation across diverse wetland soils during redox fluctuations. Using sterilized soils from 14 Chinese wetlands amended with a model methyl donor, we identified a linear relationship between hydroxyl radical (•OH) accumulation and CH_4_ production, yielding 91 nmol·L^–1^ CH_4_ per nmol·L^–1^ •OH. Mechanistic validation with citric acid and sodium citrate demonstrated that ligand-mediated iron chelation and acidification work together to enhance this pathway by preventing iron precipitation. Natural biomaterials, such as fish remnants and rice litter, acted as methyl donor hotspots, contributing approximately 50% of total CH_4_ emissions during oxygenation. These findings establish ROS-driven CH_4_ production as a pervasive abiotic pathway under ambient conditions. Our results underscore the necessity of reevaluating water management strategies in wetlands, where fluctuating water levels may inadvertently amplify abiotic CH_4_ fluxes.

## Graphical abstract

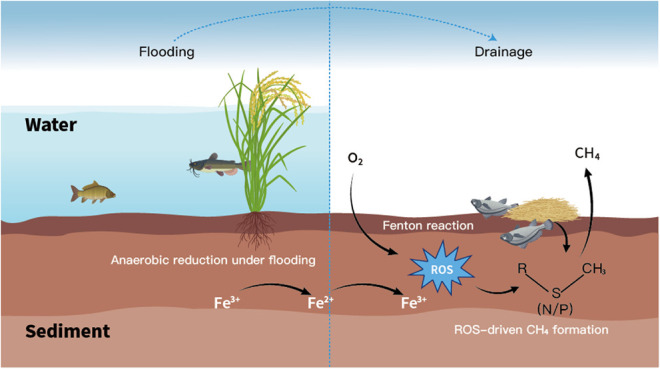

## Introduction

Methane (CH_4_) is a potent greenhouse gas, contributing to 16–25% of atmospheric warming to date.^1–3^ Approximately 40% of total global CH_4_ emits from natural sources, with wetlands being the dominant contributor.^4–6^ The expansion of wetlands due to climate change or land-use change has significantly contributed to the recent rise of CH_4_ levels.^7,8^ While anaerobic microbial pathways are recognized as dominant, the discovery of CH_4_ production under oxic conditions^9–11^ highlights gaps in our knowledge and suggests that unidentified pathways may contribute to wetland emissions.

Traditionally, CH_4_ production was considered an exclusively anaerobic microbial process, primarily occurring in anoxic wetland soils via methanogenic archaea.^12,13^ However, observations of CH_4_ oversaturation in oxic waters and soils have challenged this view.^10,11,14–18^ While some oxic CH_4_ production has been linked to microbial demethylation of specific organic compounds,^19–22^ recent breakthroughs have revealed potentially widespread abiotic pathways.^9,23–25^ Reactions involving reactive oxygen species (ROS) acting on methylated organic compounds have been identified as a source of CH_4_ across diverse biological organisms^26–29^ and potentially in abiotic environmental settings as well.^23–25^

This potential for abiotic, ROS-driven CH_4_ formation is particularly relevant to soil environments, now recognized as sites of widespread nonmicrobial greenhouse gas production.^30^ Foundational studies provided key evidence, demonstrating that dried and subsequently rewetted soils release CH_4_ chemically—a process enhanced by temperature and hydrogen peroxide.^10,11^ More recently, detailed mechanistic work elucidated the role of high-valent iron-oxo species in cleaving methyl groups from model organic substrates to generate CH_4_ precursors, even in sterilized soils at ambient temperatures.^31^ Our study connects these findings by investigating this abiotic pathway within the specific context of wetland soil reoxygenation, testing the hypothesis that endogenously generated hydroxyl radicals (•OH) from natural iron cycling are quantitatively linked to CH_4_ production across diverse wetland soils.

The ROS formed in wetland soils is abundant.^32–37^ It was found to play important roles in the detoxification of toxic elements and the degradation of organic matter in soils or sediments.^37,38^ The soil ROS is produced during the oxygenation of waterlogged soils through a Fenton-like reaction.^38^ During the reaction, oxygen reacts with the active electron acceptors in soils, particularly dissolved Fe­(II), poorly crystallized Fe­(II) species, and redox-active organic matter.^39–41^ The produced ROS is well-documented in wetland plant rhizospheres,^42,43^ sediment-water interfaces,^9^ or during the fluctuation of water tables.^34–37^ Meanwhile, nitrogen- and sulfur-containing methylated compounds are widespread in various soils and sediments.^44–49^ These compounds originate from microbial production or the breakdown of organic precursors present in detritus from phytoplankton, plants, and other organisms.^44,47^ As both ROS and methyl donor compounds are widely available in soils, the ROS-induced CH_4_ production might be significant. However, evidence for this phenomenon remains scarce, and it is unknown how it would contribute to oxic CH_4_ production.

Here, we hypothesized that methane could be abiotically formed during wetland drainage through reactions among oxygen, Fe­(II) species, and methyl donors, and that aquatic organism residues would be the hotspots of ROS-driven methane formation. We carefully investigated the factors that could influence this process, including incubation time, drainage duration, concentration of methyl donors, and the presence of organic acids. Additionally, we introduced natural materials, such as fish remnants and rice litter, into waterlogged soil columns to assess the influence of these precursors on ROS-induced CH_4_ production. Building upon established knowledge of ROS-driven CH_4_ production,^10,11,24,31^ our work bridges the gap between laboratory mechanisms and real-world fluxes. By investigating the specific role of •OH during the reoxygenation of wetland soils, we provide quantitative evidence extending the •OH-induced CH_4_ formation pathway to wetland soils under ambient conditions.

## Materials and Methods

### Site Selection and Soil Characterization

Soil samples were systematically collected across a latitudinal and climatic gradient in China to capture biogeochemical variability across major terrestrial ecosystems (Table S1). Sites were selected to represent contrasting pedogenic regimes, encompassing variations in climatic parameters and soil textures. Physicochemical properties were quantified using standardized protocols (Table S1).

### Redox Fluctuation Microcosm Experiments

#### Anaerobic Preincubation

Wetland soil slurries were prepared under strict anoxic conditions to simulate natural reducing environments. Triplicate aliquots (15.0 ± 0.1 g dry weight equivalent) were homogenized with 60 mL of Milli-Q water (1:4 soil-to-solution ratio) in 125 mL serum bottles, sealed with butyl rubber septa (Figure S5), and subjected to three alternating cycles of vacuum-purge with ultrapure N_2_ (99.999%, 1 atm). Bottles were incubated in the dark at 25 ± 1 °C for 15 days within an anaerobic chamber (Vigor IG1200/750TS; atmosphere: 95% N_2_/5% H_2_; Pd-catalyzed O_2_ scrubbing <5 ppm), with continuous orbital shaking (220 rpm) to enhance biogeochemical homogeneity.

Postincubation: biological activity was terminated via pasteurization (85 °C, 12 h). Residual CH_4_ was stripped via three sequential headspace replacements with N_2_, as verified by gas chromatography (GC) baseline stabilization. ROS generation was initiated by injecting 50 mL O_2_ (99.995%) and 100 mM dimethyl sulfoxide (DMSO, as a methyl precursor) through septa using gastight syringes (Hamilton). Microcosms were agitated (120 rpm, 25 °C) under light-restricted conditions to preclude photochemical artifacts. Headspace CH_4_ (0.5 mL) and slurry aliquots (1 mL) were sampled at 0, 12, 24, 48, and 72 h for parallel gas and solid-phase analyses.

### Organic Substrate Treatments

To evaluate the methyl precursor efficacy and partition CH_4_ sources, incubations were amended with synthetic or natural organic substrates. DMSO served as the primary model S-methyl substrate, given its known reactivity in iron-mediated abiotic oxidation. Dimethyl sulfide (DMS) and dimethylsulfoniopropionate (DMSP), intermediates in environmental sulfur cycling, were included for comparison. Synthetic substrates were added to a final concentration of 100 mM immediately before oxygenation. For natural substrates, crucian carp (*Carassius carassius*) tissue was freeze-dried, while rice (*Oryza sativa*) straw and leaves were rinsed, oven-dried (60 °C, 48 h), ground, and sieved (<1 mm). These processed natural materials were amended to soil slurries at 0.1% or 1% (w/w) prior to anaerobic preincubation, during which they were microbially processed into methyl-containing compounds. In addition, to investigate the effect of low-molecular-weight organic acids (LMWOA) on ROS-driven CH4 production in soil, we used citric acid (CA) as a model compound. A subset of soil slurries was treated with 6.25 mM CA and 100 mM DMSO, with a control treated with DMSO alone. The addition of LMWOA, such as CA, results in a lower pH and promotes chelation simultaneously, both of which facilitate Fenton-type ROS production. Therefore, we also used sodium citrate at the same concentration as a parallel treatment. This approach ensures that the ligand concentration is the same as in the CA treatment while avoiding a reduction in pH, allowing us to differentiate the effects of pH changes from those of ligand-mediated iron chelation.

### Drainage Simulation Microcosms

A custom-designed column system equipped with a bottom outlet valve (borosilicate glass; 15 cm height × 10 cm diameter) was employed to replicate paddy soil drainage dynamics (Figure S6). The columns contained 150 g of air-dried soil (sieved ≤ 2 mm), 300 mL of deionized water, and 10 g of fish remnants. After a 25-day anoxic incubation (25 °C, dark), the incubated soil columns were divided into three treatments. A subset of columns remained unsterilized, while the remaining columns were sterilized by pasteurization (85 °C, 12 h) to terminate biological activity. Drainage was simulated by opening the bottom valve to discharge both the overlying water and the soil porewater. To balance the pressure and maintain anoxic conditions, the headspace gas was simultaneously replaced with N_2_ by flushing through injection needles inserted through the rubber septa during the drainage process. Immediately following drainage, the headspace of the oxygenation treatment groups was flushed with ambient air to establish oxic conditions, while the anoxic control groups were maintained under N_2_ (Figure 5). This resulted in three treatments: (1) unsterilized columns receiving O_2_ in the headspace, (2) sterilized columns receiving O_2_ in the headspace, and (3) sterilized columns maintained under an N_2_ headspace. Headspace CH_4_ concentrations were measured immediately after flushing (0 h) and again after 48 h of incubation.

### Hydroxyl Radical Quantification

•OH production was tracked via benzoate dosimetry. Soil slurries (15 g + 60 mL of 100 mM sodium benzoate) were incubated under experimental O_2_ regimes. At 0–72 h intervals, 1 mL aliquots were quenched in methanol (1:1 v/v), filtered (0.22 μm PTFE), and analyzed for para-hydroxybenzoate (p-HBA) via HPLC-UV (Agilent 1260; Eclipse Plus C18 column; 0.1% TFA/acetonitrile mobile phase; 254 nm). Cumulative •OH concentration was calculated using a stoichiometric conversion factor (5.87 ± 0.12 μmol •OH per μmol p-HBA). This factor accounts for the yields of different hydroxylated products and has been previously applied and validated for quantifying •OH generation in sediment oxygenation studies.^32,50^

### Gas Chromatography

CH_4_ was quantified via GC-FID (Agilent 7890B) with a PLOT-Q column (30 × 0.32 mm). Method parameters: injector: 200 °C; FID: 250 °C; carrier gas (He): 2.5 mL min^–1^; oven program: 100 °C (2.5 min) → 190 °C at 30 °C min^–1^ (0.5 min hold). Calibration utilized certified CH_4_ standards (5–500 ppmv; Restek).

### Iron Speciation Determination

HCl-extractable Fe­(II/III) was determined via a colorimetric assay. Slurries (1 mL) were extracted in 0.5 M HCl (5 mL, 16 h, 120 rpm) and centrifuged (3,000 × *g*, 15 min). Fe­(II) in the supernatant was quantified directly against ferrous ethyl sulfate standards using the 0.1% phenanthroline method, with absorbance measured spectrophotometrically at 512 nm using a microplate reader (Tecan Spark 20M). Total iron was determined similarly after the reduction of Fe­(III) to Fe­(II) using 10% hydroxylamine. Fe­(III) concentration was calculated as the difference between total iron and Fe­(II).

## Results and Discussion

### Abiotic CH_4_ Formation in Diverse Soils

Soil samples were collected from 14 paddy field and wetland sites across China (Figure 1a, Table S1). After initial flooding and sterilization, all tested samples were able to produce CH_4_ upon oxygenation with dimethyl sulfoxide (DMSO) addition (Figure 1c,d). Despite the variability in CH_4_ production potential among the sites, no clear geographic pattern was observed (Table S2). A positive correlation was observed between •OH accumulation and CH_4_ concentrations after 360 h of oxygenation, with a slope of 0.091 μmol·L^–1^ per nmol·L^–1^ (*R* ^2^ = 0.58, *p* = 0.0016) (Figure 1b). The abiotic demethylation process is likely complex, as reflected by the moderate correlation strength. Measured •OH represents the Fenton-like aspect, but direct oxidation by unmeasured high-valent iron-oxo species probably also contributes substantially to methyl radical (•CH_3_) generation.^31^

**Figure 1:**
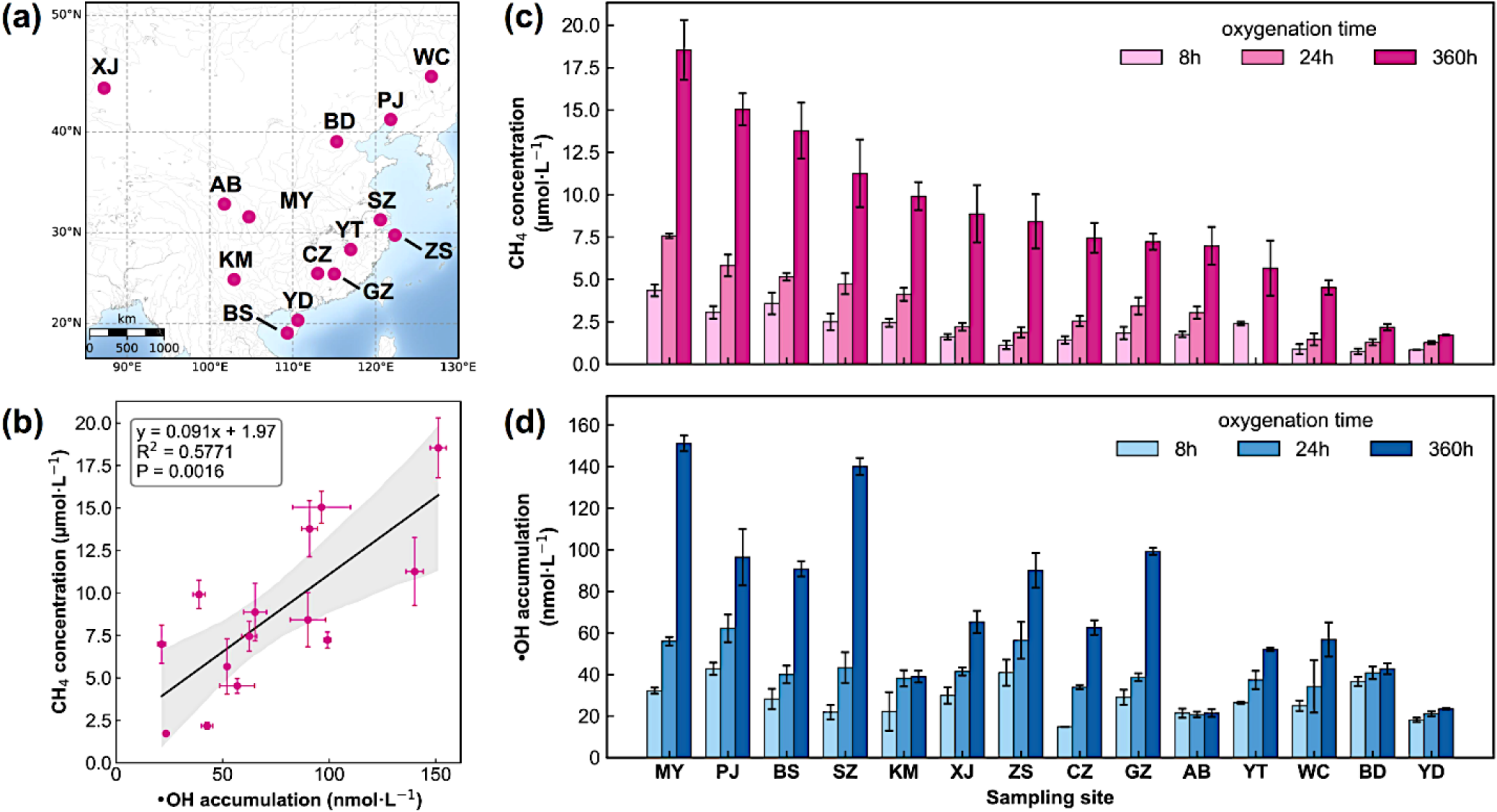
Abiotic CH_4_ production and •OH accumulation during the oxygenation of anoxic soil slurries amended with 100 mM DMSO. (a) Geographical distribution of the 14 wetland sampling sites across China. (b) Linear regression between •OH accumulation and CH_4_ concentration after 360 h of oxygenation (*n* = 14). The shaded area indicates the 95% confidence interval. (c) Time course of CH_4_ concentrations (μmol·L^–1^) and (d) •OH accumulation (nmol·L^–1^) at 8, 24, and 360 h. Sites are arranged in descending order of CH_4_ yield at 360 h. Sampling sites are denoted by abbreviations: BS (Baisha), YD (Yuedong), BD (Baoding), PJ (Panjin), WC (Wuchang), CZ (Chenzhou), SZ (Suzhou), GZ (Ganzhou), YT (Yantai), AB (Aba), MY (Mianyang), XJ (Xinjiang), KM (Kunming), and ZS (Zhoushan). Bars represent means ± s.d. from triplicates (*n* = 3).

The CH_4_ concentrations ranged from 1.12 μmol·L^–1^ in YD soils to over 121.57 μmol·L^–1^ in MY soils (Figure 1c). In most samples, both CH_4_ and •OH levels increased with oxygenation time, except for two regions exhibiting low CH_4_ production, where the •OH levels remained relatively insensitive to oxygenation. Overall, our findings demonstrate that ROS-driven CH_4_ production is a ubiquitous phenomenon in wetland soils when amended with the model methyl donor DMSO. Nonetheless, the ultimate abiotic CH_4_ yield appears to be modulated by multiple factors, including the availability of Fe­(II) species and methyl donor compounds.

### CH_4_ Production Influenced by ROS Accumulation

According to the hypothesis that abiotic CH_4_ formation is driven by ROS generated through the interaction of Fe­(II) species and oxygen, we assumed that extending the flooding and drainage periods would influence Fe­(II) cycling, thereby enhancing ROS accumulation and CH_4_ production. As the anoxic incubation time increased, the extent of reduction in the lower-crystalline mineral iron also increased (Figure S4a). When soils were flooded for 1 to 16 days, the extent of Fe­(II) oxidation after 72 h of oxygenation increased with longer flooding durations, showing a rapid rise from day 1 to day 13, followed by a plateau (Figure 2a). Similarly, •OH accumulation showed a linear increase with flooding duration up to day 11, after which it fluctuated slightly (Figure 2a,b and Figure S2). In parallel, the CH_4_ concentration in the headspace increased linearly with the flooding duration (Figure 2b), indicating that prolonged flooding enhances CH_4_ formation upon subsequent oxygenation. In our experiment, sterilization removed the contribution of microbes to methane production but also inactivated iron-reducing bacteria. Therefore, we were unable to simulate the wet–dry cycles. However, in natural environments, hotspots of iron cycling and reactive oxygen species driven by wet–dry cycles are known to exist. We can therefore believe that the abiotic methane production process observed in the experiment could also occur alongside wet–dry cycling.

**Figure 2:**
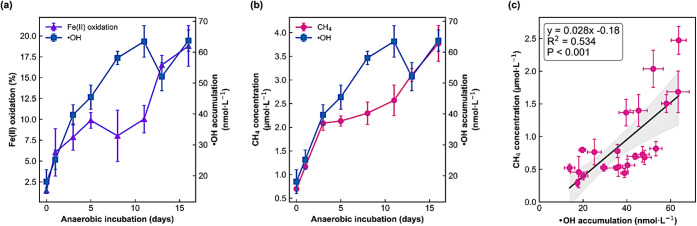
Effect of anaerobic incubation duration (0–16 days) on Fe­(II) oxidation, •OH accumulation, and CH_4_ production in ZS soil slurries after 72 h of oxygenation. (a) Extent of Fe­(II) oxidation and •OH accumulation plotted against the duration of the preceding anaerobic incubation. (b) CH_4_ concentration and •OH accumulation across the same incubation periods. (c) Linear regression analysis showing a significant positive correlation between •OH accumulation and CH_4_ concentration (*n* = 22). Error bars represent means ± s.d. from triplicates (*n* = 3). The shaded region in (c) indicates the 95% confidence interval.

Oxygenation experiments were conducted to simulate the effects of drainage, during which CH_4_ and •OH accumulation were monitored at different oxygenation times. During the oxygenation process, a rapid surge in •OH generation was observed within the first 10 h, followed by a more gradual accumulation over the remaining 62 h of the 72 h period (Figure S3a). Concurrently, the reduction in the 0.5 M HCl-Fe­(II) proportion increased steadily (Figure S3b), reflecting the role of Fe­(II) oxidation in driving •OH generation. In contrast, CH_4_ formation exhibited a steadier trend, increasing throughout the 72-h oxygenation period (Figure S3c). The increase in the level of ROS at all incubation time points was time dependent, although the total ROS production varied. The maximum CH_4_ concentration in the headspace, approximately 3.77 μmol·L^–1^, was detected in soils that had been flooded for 16 days and oxygenated for 72 h. A positive correlation between CH_4_ production and •OH accumulation (Figure 2c) highlights the critical role of hydroxyl radicals in driving CH_4_ formation. These findings reveal the intricate interplay among Fe­(II) oxidation, ROS generation, and CH_4_ production, with •OH emerging as a key driver of CH_4_ formation in oxygenated soils (Figure S4).

### CH_4_ Production Influenced by Methyl Donors

Methyl donors are a critical limiting factor in soil-ROS-induced CH_4_ generation. Methane formation nearly doubled when the DMSO addition increased from 0.1 to 1 mM and again doubled when the addition increased from 1 to 5 mM (Figure 3a). These results highlight the strong role of DMSO as a methyl donor in driving CH_4_ production. In contrast, other chemical precursors, such as DMS and DMSP, were far less effective, inducing only weak CH_4_ formation (Figure 4b).

**Figure 3:**
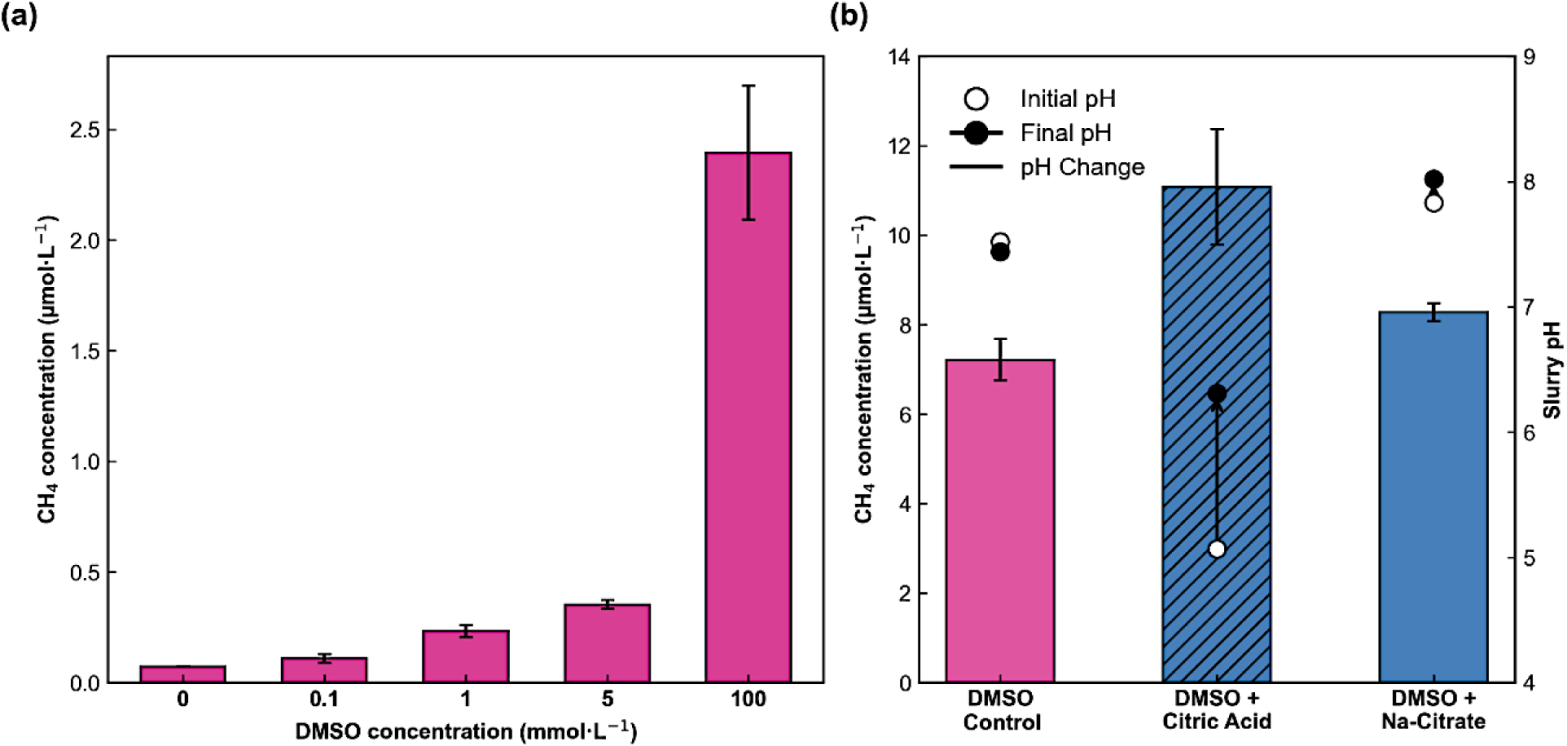
Influence of methyl donor concentration and iron-modulating agents on abiotic CH_4_ production in ZS soil slurries over a 5-day oxygenation period. (a) CH_4_ accumulation in response to increasing initial concentrations of DMSO (0–100 mM). (b) Mechanistic comparison of CH_4_ production and pH changes in slurries amended with 100 mM DMSO alone, DMSO + 6.25 mM citric acid (CA), or DMSO + 6.25 mM sodium citrate (Na-Citrate). The pH data distinguish the synergistic effect of acidification and chelation (CA) from ligand-mediated chelation alone (Na-Citrate). Error bars represent means ± s.d. from biological replicates (*n* = 3).

**Figure 4:**
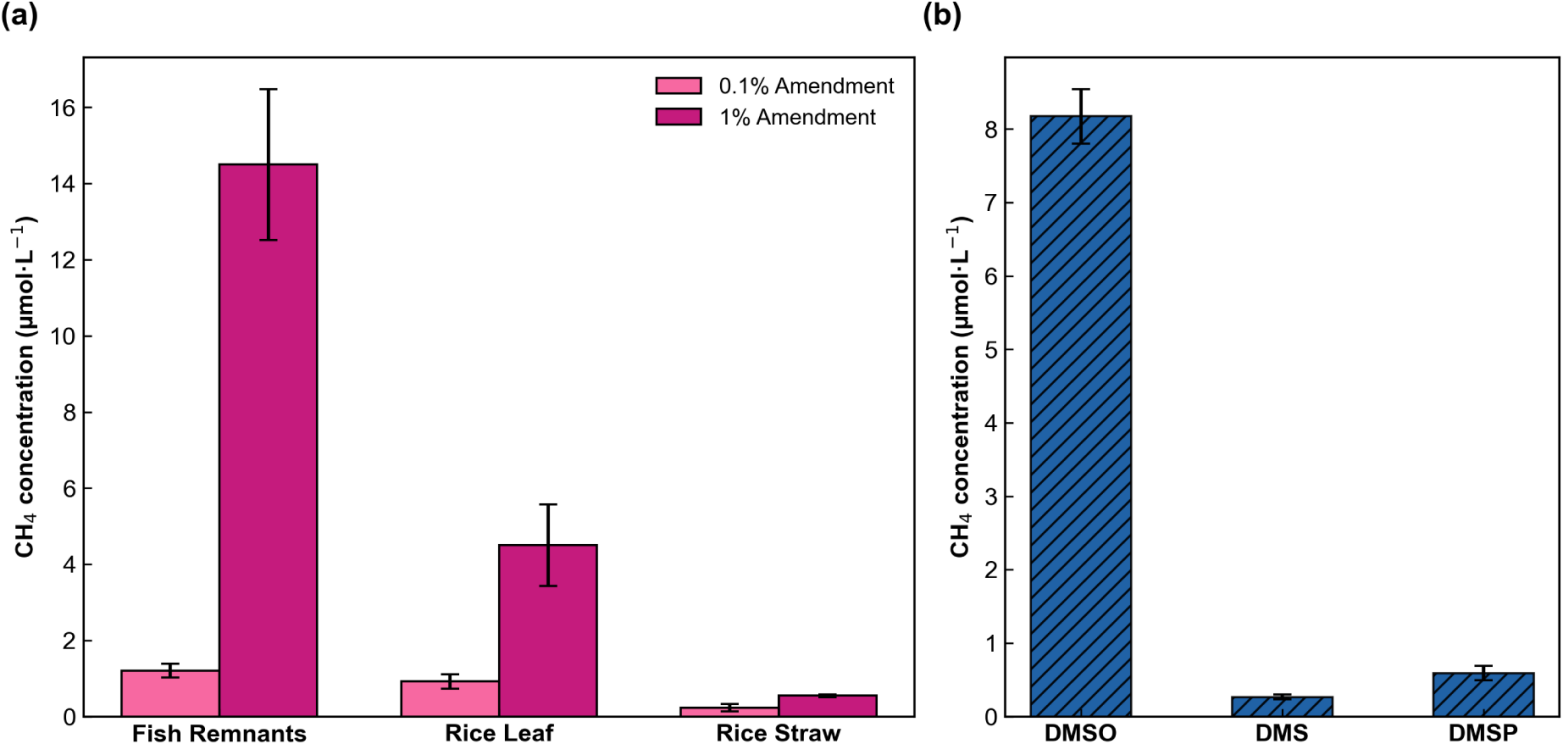
CH_4_ production from natural and synthetic precursors during 15 days of oxygenation in anoxic Zhoushan (ZS) soil slurries. (a) Dose-dependent CH_4_ accumulation in slurries amended with natural organic matter (fish remnants, rice leaves, and rice straw) at low (0.1% w/w) and high (1% w/w) dosages. (b) CH_4_ production from synthetic organic substrates (100 mM), including DMSO, DMS, and DMSP. Error bars represent means ± s.d. from biological replicates (*n* = 3).

We further tested the potential of varying natural organic matter amendments to generate CH_4._ CH_4_ yields varied significantly among treatments and showed a clear dose-dependent response (Figure 4a). Increasing the amendment dosage from 0.1% to 1% (w/w) consistently amplified CH_4_ accumulation across all substrates. Animal-derived organic matter, specifically fish remnants, was the most effective CH_4_ precursor, producing the highest CH_4_ levels at the 1% dosage. This is likely due to the abundance of sulfur- and nitrogen-bonded methyl groups in protein-rich tissues, which are readily oxidized to drive ROS-induced CH_4_ formation.

Among plant-based sources, rice leaves showed moderate CH_4_ production, while rice straw yielded lower but detectable CH_4_ emissions. While the 0.1% straw amendment produced minimal methane, the 1% dosage resulted in a distinct increase, confirming that even recalcitrant agricultural residues can contribute to abiotic CH_4_ production at higher accumulation levels. These results suggest that animal-derived organic matter could act as a hotspot for ROS-induced CH_4_ production in flooded soils upon drainage, while plant litter contributes a smaller but widespread background flux. The substantial differences between plant- and animal-derived organic matter emphasize the varying biochemical composition of these sources and their implications for CH_4_ production dynamics.^18^

### Role of pH and Iron Chelation in Oxic CH_4_ Production

To explore the potential effects of LWMOA on abiotic CH_4_ production during oxygenation, we selected CA as a representative organic acid commonly occurring in wetland soils. In our batch experiments, the addition of CA led to the highest accumulation of CH_4_ during oxygenation, demonstrating a significant enhancement compared to the control treatment with DMSO alone (Figure 3b). It is well established that LWMOA facilitates Fenton-type reactions by lowering soil pH and forming complexes with Fe­(II) and Fe­(III), which prevent the precipitation of iron oxides and increase the concentration of available iron. Our findings substantiated that the promoting effects of LWMOA on soil ROS formation under oxic conditions further contributed to enhanced abiotic CH_4_ production in the presence of methylated organosulfur compounds. Both citric acid and sodium citrate stimulated ROS generation and CH_4_ formation, consistent with ligand-mediated iron mobilization playing a central role in this process. The addition of citric acid caused an immediate decrease in the slurry pH, which returned to near-neutral conditions within 24 h due to soil buffering.

To disentangle the relative contributions of acidification and iron chelation, sodium citrate was used to isolate the effect of ligand-mediated chelation without inducing acidity. Despite the absence of acidification, sodium citrate still significantly increased CH_4_ production compared with the control, resulting in an approximately 14% enhancement attributable to chelation alone.

By comparing the two citrate treatments, the overall enhancement observed with citric acid addition can be approximated as comprising a chelation-related component and an additional enhancement associated with short-term acidification. Relative to the control, sodium citrate accounted for approximately 26% of the total increase in CH_4_ production, representing the contribution of ligand-mediated iron chelation alone. The remaining ∼74% of the enhancement observed in the citric acid treatment reflects the combined effect of iron chelation and short-term acidification occurring during the initial reaction stage.

Given the buffering capacity of soils, organic acid inputs in natural environments are expected to induce brief pH depressions. These are subsequently buffered toward near-neutral conditions, while citrate persists predominantly in deprotonated forms capable of chelating Fe­(II) and Fe­(III). In this context, the citric acid and sodium citrate treatments can be viewed as approximating successive stages of a continuous process. Transient acidification induces a short-lived but high-intensity increase in ROS generation beyond chelation alone, whereas chelation-driven iron cycling supports lower-level but sustained ROS production over longer time scales, cumulatively promoting abiotic CH_4_ formation.

### ROS-Induced CH_4_ Generation After Drainage

To evaluate the contribution of •OH-induced CH_4_ production under conditions mimicking field drainage, we simulated a drainage event using soil columns containing fish remnants after a 25-day anoxic incubation (Figure 5). Following incubation, columns were either sterilized or left unsterilized prior to drainage treatments, enabling the separation of abiotic CH_4_ production from combined biotic–abiotic processes. Drainage was simulated by removing porewater and replacing the column headspace with either ambient air or N_2_, after which the columns were sealed and incubated. Three treatments were thus established: unsterilized columns with an oxygenated headspace, sterilized columns with an oxygenated headspace, and sterilized columns maintained under an anoxic N_2_ headspace. When ambient air was introduced into the headspace of the sterilized column (Sterilized + O_2_ Drainage), approximately 11.78 μmol·L^–1^ CH_4_ accumulated over 48 h. This abiotic production accounted for roughly half of the total CH_4_ measured in the unsterilized column exposed to air (Unsterilized + O_2_ Drainage). In contrast, the sterilized column kept under an anoxic N_2_ headspace (Sterilized + N_2_ Drainage) produced negligible CH_4_, confirming the essential role of oxygen in driving this abiotic formation process.

**Figure 5:**
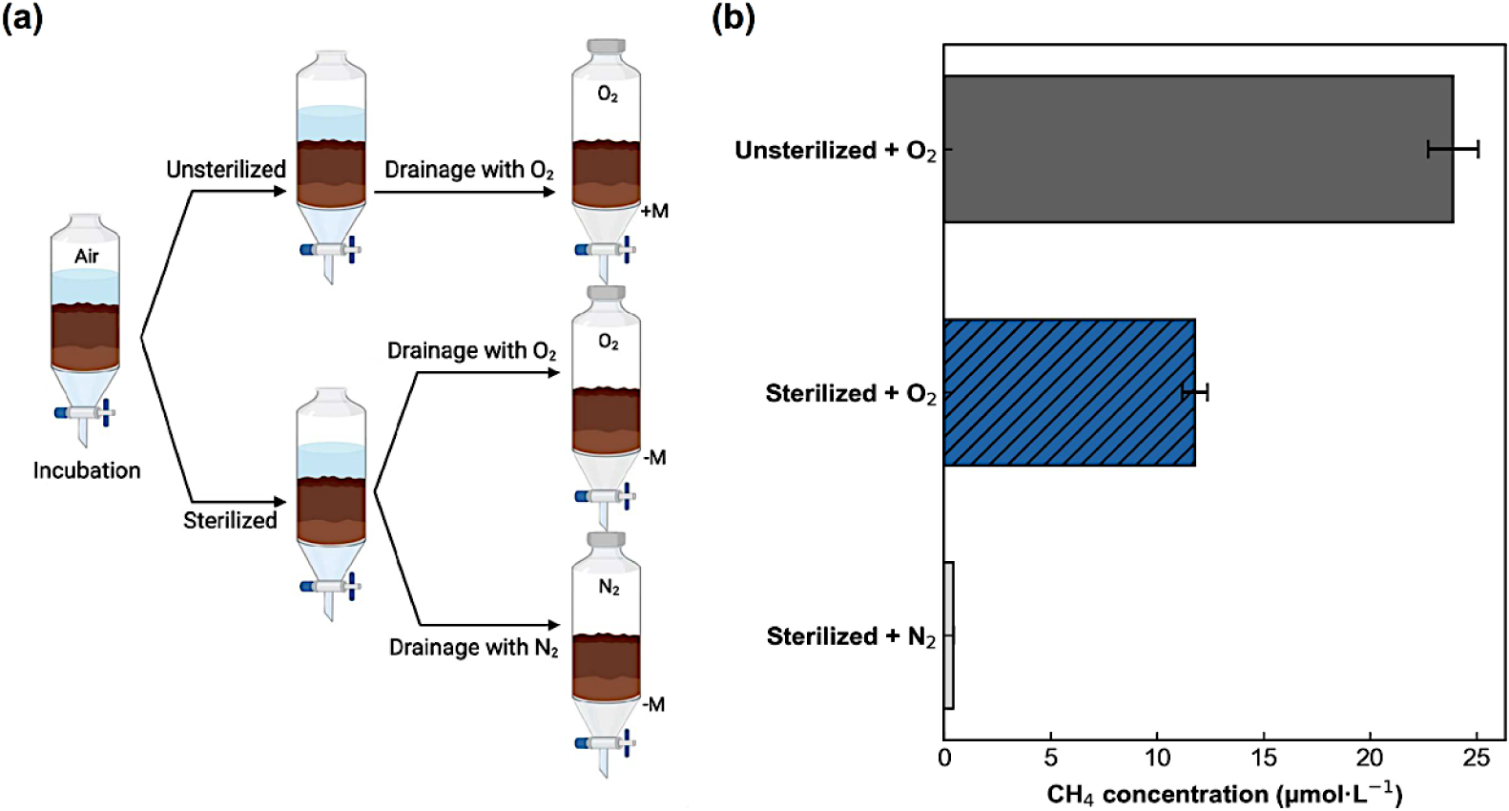
Contributions of biotic and abiotic processes to CH_4_ production under simulated drainage. CH_4_ production was evaluated under three distinct treatments after a 25-day anoxic incubation with fish remnant: (1) Unsterilized + O_2_ Drainage: Nonsterile soil column with headspace exposed to ambient air; (2) Sterilized + O_2_ Drainage: Sterilized soil column with headspace exposed to ambient air; and (3) Sterilized + N_2_ Drainage: Sterilized soil column with headspace purged with N_2_. Error bars represent the standard deviation from triplicate measurements (*n* = 3).

These results suggest a potentially significant contribution of abiotic, ROS-driven pathways to CH_4_ fluxes during soil drainage events. However, several factors inherent to the experimental design influence this interpretation. Oxygen transfer from the headspace into the saturated soil column is diffusion-limited, likely creating a temporal redox gradient rather than immediate, uniform oxic conditions. This gradual shift could influence abiotic reaction rates and potentially allow the temporary persistence of anaerobic microbial processes. Furthermore, potential methane consumption by methanotrophs stimulated by oxygen ingress in the unsterilized column was not quantified. Consequently, our estimate that abiotic processes contribute approximately 50% should be viewed as an approximation under these specific experimental conditions. Nonetheless, the observed CH_4_ burst upon oxygenation provides a potential mechanistic explanation involving ROS generation for earlier findings where rewetting of dried soils significantly enhanced CH_4_ emissions.^10,11^

## Environmental Implications

Our study uncovers a major abiotic pathway for CH_4_ generation in wetland soils, mediated by •OH produced during redox fluctuations. This mechanism challenges the conventional focus on microbial methanogenesis and redefines our understanding of CH_4_ sources in waterlogged ecosystems. Our work builds upon recent studies that established the chemical basis for iron-mediated abiotic CH_4_ formation at ambient and elevated temperatures while quantifying ROS-driven CH_4_ production (91 nM CH_4_ per nM •OH) across 14 diverse Chinese wetlands. By systematically linking this production to •OH accumulation across diverse wetland soils during simulated reoxygenation and drainage, we extend the foundational observations from earlier soil studies and provide quantitative evidence for the pathway’s significance in a dynamic environmental context. This •OH-mediated abiotic CH_4_ production is not confined to wetlands. The Fe­(II) oxidation that generates •OH radicals is prevalent in various environments, including coastal sediments,^33^ rhizospheres,^42,43^ detritusphere,^51^ forest soils,^52^ and rice paddies^53,54^ where redox fluctuations and methyl donors coexist.^11,44,47^ Indeed, our findings help clarify prior field and laboratory observations where methane presence under oxic conditions during redox cycling experiments in paddy soils was noted but not fully explained.^54^ Our work suggests that such observations could be attributed to the •OH-mediated decomposition of soil organic matter constituents acting as methyl donors, a pathway not previously identified in that context.

A key mechanistic question is the fate of the •CH_3_ intermediate. In oxic systems, •CH_3_ is expected to react rapidly with O_2_. The formation of CH_4_ requires the competing reaction of hydrogen atom abstraction, implying that sufficient hydrogen donors, likely from the soil organic matter matrix itself, must be available—a condition noted in similar iron-based systems. This suggests that the total demethylation rate may be significantly higher than the rate of CH_4_ formation.

The rise in atmospheric CH_4_ levels, often linked to wetland expansion under climate change,^8,55^ may now also reflect abiotic contributions. While microbial processes dominate current models,^56^ ROS-driven CH_4_ emissions could amplify as climate change intensifies hydrological extremes. It is also important to acknowledge that our current understanding of biotic methane production might be underestimated. This is because potential methane oxidation has not been thoroughly accounted for in our assessments.^57,58^ For instance, wetland degradation and frequent flooding-drainage cycles enhance Fe­(II) availability, fueling •OH generation, and potentially subsequent CH_4_ production. Water management strategies like alternate wetting and drying (AWD), designed to curb microbial CH_4_ in rice paddies, risk unintended consequences: prolonged flooding phases accumulate Fe­(II), and subsequent drainage triggers abiotic CH_4_ bursts via •OH-driven demethylation of organic matter (e.g., fish remnants or rice litter). Shorter oxic intervals in flooded soils enhance Fe reduction, while longer oxic intervals favor CH_4_ emissions.^48^ This pattern exemplifies the complex relationship among redox dynamics, microbial activity, and CH_4_ production.

These insights demand optimization of wetland management—such as fine-tuning drainage timing or limiting methyl donor accumulation—that could curb abiotic CH_4_ without sacrificing agricultural productivity. Furthermore, the interplay between biotic and abiotic pathways (e.g., Fenton-like reactions in upland soils) warrants deeper exploration to refine emission inventories. Addressing these gaps is critical for accurate climate projections and policies, particularly as warming accelerates hydrological disruptions.

## Supplementary Material



## Notes

Table of soil sample locations and physicochemical properties (Table S1); Table of CH _4_ and •OH production data for all 14 soil samples (Table S2); Figures showing CH_4_ production under varied substrate conditions, temporal trends in •OH, Fe­(II), and CH_4_, and experimental workflow schematics (Figures S1–S6) (PDF)

## Abbreviations

CA: Citric Acid
DMS: Dimethyl Sulfide
DMSP: Dimethyl Sulfoniopropionate
DMSO: Dimethyl Sulfoxide
GC-FID: Gas Chromatography-Flame Ionization Detection
HPLC-UV: High-Performance Liquid Chromatography-Ultraviolet
p-HBA: para-hydroxybenzoate
ROS: Reactive Oxygen Species

## References

[1] Lashof D., Ahuja D. (1990). Relative contributions of greenhouse gas emissions to global warming. Nature.

[2] Badr O., Probert S. D., O’Callaghan P.
W. (1991). Atmospheric CH_4_: Its contribution to global warming. Appl.
Energy.

[3] Rosentreter J. A., Borges A. V., Deemer B. R., Holgerson M. A., Liu S., Song C., Melack J., Raymond P. A., Duarte C. M., Allen G. H. (2021). Half of global methane emissions come from
highly variable aquatic ecosystem sources. Nat.
Geosci..

[4] Zhang Z., Zimmermann N. E., Stenke A., Li X., Hodson E. L., Zhu G., Huang C., Poulter B. (2017). Emerging role of wetland CH_4_ emissions in driving 21st century climate change. Proc. Natl. Acad. Sci. U. S. A..

[5] Bridgham S. D., Cadillo-Quiroz H., Keller J. K., Zhuang Q. (2013). CH_4_ emissions
from wetlands: Biogeochemical, microbial, and modeling perspectives
from local to global scales. Global Change Biol..

[6] Zhang B., Tian H., Ren W., Tao B., Lu C., Yang J., Banger K., Pan S. (2016). CH_4_ emissions
from global rice fields: Magnitude, spatiotemporal patterns, and environmental
controls. Global Biogeochem. Cycles.

[7] Nikolaisen M., Cornulier T., Hillier J., Smith P., Albanito F., Nayak D. (2023). CH_4_ emissions from rice paddies globally: A quantitative
statistical review of controlling variables and modelling of emission
factors. J. Clean. Prod..

[8] Zhang Z., Poulter B., Feldman A. F., Ying Q., Ciais P., Peng S., Li X. (2023). Recent intensification
of wetland
CH_4_ feedback. Nat. Clim. Chang..

[9] Ye J., Hu A., Gao C., Li F., Li L., Guo Y., Ren G., Li B., Rensing C., Nealson K. H., Zhou S., Xiong Y. (2024). Abiotic methane
production driven by ubiquitous non-Fenton-type reactive
oxygen species. Angew. Chem., Int. Ed..

[10] Hurkuck M., Althoff F., Jungkunst H. F., Jugold A., Keppler F. (2012). Release of
methane from aerobic soil: An indication of a novel chemical natural
process?. Chemosphere.

[11] Jugold A., Althoff F., Hurkuck M., Greule M., Lenhart K., Lelieveld J., Keppler F. (2012). Non-microbial methane formation in
oxic soils. Biogeosciences.

[12] Liu Y., Whitman W. B. (2008). Metabolic, phylogenetic,
and ecological diversity of
the methanogenic archaea. Ann. N.Y. Acad. Sci..

[13] Segers R. (1998). CH_4_ production and CH_4_ consumption:
a review of processes
underlying wetland CH_4_ fluxes. Biogeochemistry.

[14] Donis D., Flury S., Stöckli A., Spangenberg J. E., Vachon D., McGinnis D. F. (2017). Full-scale evaluation
of CH_4_ production under oxic conditions in a mesotrophic
lake. Nat. Commun..

[15] Günthel M., Donis D., Kirillin G., Ionescu D., Bizic M., McGinnis D. F., Grossart H.-P., Tang K. W. (2019). Contribution
of
oxic CH_4_ production to surface CH_4_ emission
in lakes and its global importance. Nat. Commun..

[16] Schroll M., Liu L., Einzmann T., Keppler F., Grossart H. P. (2023). CH_4_ accumulation
and its potential precursor compounds in the oxic surface water layer
of two contrasting stratified lakes. Sci. Total
Environ..

[17] Grossart H. P., Frindte K., Dziallas C., Eckert W., Tang K. W. (2011). Microbial
CH_4_ production in oxygenated water column of an oligotrophic
lake. Proc. Natl. Acad. Sci. U. S. A..

[18] Angle J. C., Morin T. H., Solden L. M., Narrowe A. B., Smith G. J., Borton M. A., Rey-Sanchez C., Daly R. A., Mirfenderesgi G., Hoyt D. W., Riley W. J., Miller C. S., Bohrer G., Wrighton K. C. (2017). Methanogenesis in
oxygenated soils is a substantial
fraction of wetland methane emissions. Nat.
Commun..

[19] Karl D. M., Beversdorf L., Björkman K. M., Church M. J., Martinez A., DeLong E. F. (2008). Aerobic
production of CH_4_ in the sea. Nat.
Geosci..

[20] von
Arx J. N., Kidane A. T., Philippi M., Mohr W., Lavik G., Schorn S., Kuypers M. M. M., Milucka J. (2023). Methylphosphonate-driven
CH_4_ formation and its link to primary production in the
oligotrophic North Atlantic. Nat. Commun..

[21] Zheng Y., Harris D. F., Yu Z., Fu Y., Poudel S., Ledbetter R. N., Fixen K. R., Yang Z.-Y., Boyd E. S., Lidstrom M. E., Seefeldt L. C., Harwood C. S. (2018). A pathway
for biological
CH_4_ production using bacterial iron-only nitrogenase. Nat. Microbiol..

[22] North J. A., Narrowe A. B., Xiong W., Byerly K. M., Zhao G., Young S. J., Murali S., Wildenthal J. A., Cannon W. R., Wrighton K. C. (2020). A nitrogenase-like enzyme
system catalyzes methionine, ethylene, and methane biogenesis. Science.

[23] Ernst L., Barayeu U., Hädeler J., Dick T. P., Klatt J. M., Keppler F., Rebelein J. G. (2023). CH_4_ formation driven by
light and heat prior to the origin of life and beyond. Nat. Commun..

[24] Ernst L., Steinfeld B., Barayeu U., Klintzsch T., Kurth M., Grimm D., Dick T. P., Rebelein J. G., Bischofs I. B., Keppler F. (2022). CH_4_ formation driven by
reactive oxygen species across all living organisms. Nature.

[25] Althoff F., Benzing K., Comba P., McRoberts C., Boyd D. R., Greiner S., Keppler F. (2014). Abiotic methanogenesis
from organosulphur compounds under ambient conditions. Nat. Commun..

[26] Lenhart K., Bunge M., Ratering S., Neu T. R., Schüttmann I., Greule M., Kammann C., Schnell S., Müller C., Zorn H. (2012). Evidence
for methane production by saprotrophic fungi. Nat. Commun..

[27] Klintzsch T., Langer G., Nehrke G., Wieland A., Lenhart K., Keppler F. (2019). CH_4_ production by three widespread marine
phytoplankton species: release rates, precursor compounds, and potential
relevance for the environment. Biogeosciences.

[28] Bižić M., Klintzsch T., Ionescu D., Hindiyeh M. Y., Günthel M., Muro-Pastor A. M., Eckert W., Urich T., Keppler F., Grossart H.-P. (2020). Aquatic and terrestrial cyanobacteria produce CH_4_. Sci. Adv..

[29] Hilt S., Grossart H. P., McGinnis D. F., Keppler F. (2022). Potential role of submerged
macrophytes for oxic CH_4_ production in aquatic ecosystems. Limnol. Oceanogr..

[30] Wang B., Lerdau M., He Y. (2017). Widespread
production of non-microbial
greenhouse gases in soils. Global Change Biol..

[31] Hädeler J., Velmurugan G., Lauer R., Radhamani R., Keppler F., Comba P. (2023). Natural Abiotic
Iron-Oxido-Mediated
Formation of C_1_ and C_2_ Compounds from Environmentally
Important Methyl-Substituted Substrates. J.
Am. Chem. Soc..

[32] Tong M., Yuan S., Ma S., Jin M., Liu D., Cheng D., Liu X., Gan Y., Wang Y. (2016). Production
of Abundant Hydroxyl Radicals from Oxygenation of Subsurface Sediments. Environ. Sci. Technol..

[33] Zhao G., Wu B., Zheng X., Chen B., Kappler A., Chu C. (2022). Tide-triggered
production of reactive oxygen species in coastal soils. Environ. Sci. Technol..

[34] Jia M., Bian X., Yuan S. (2017). Production of hydroxyl radicals from
Fe­(II) oxygenation induced by groundwater table fluctuations in a
sand column. Sci. Total Environ..

[35] Zhang Y., Zhang N., Yu C., Liu H., Yuan S. (2023). ROS production
upon groundwater oxygenation: Implications of oxidative capacity during
groundwater abstraction and discharging. J.
Hydrol..

[36] Liu F., Wang Z., Liu J., Latif J., Qin J., Yang H., Jiang W., Deng Y., Yang K., Ni Z. (2024). Seasonal
and spatial fluctuations of reactive oxygen
species in riparian soils and their contributions on organic carbon
mineralization. Environ. Sci. Technol..

[37] Du H., Wang H., Chi Z., Song N., Wang C., Xu H. (2021). Burst of hydroxyl radicals in sediments derived by flooding/drought
transformation process in Lake Poyang, China. Sci. Total Environ..

[38] Huang D., Chen N., Zhu C., Sun H., Fang G., Zhou D. (2023). Dynamic production of hydroxyl radicals during the flooding-drainage
process of paddy soil: An in situ column study. Environ. Sci. Technol..

[39] Yu C., Zhang Y., Lu Y., Qian A., Zhang P., Cui Y., Yuan S. (2021). Mechanistic insight into humic acid-enhanced hydroxyl
radical production from Fe­(II)-bearing clay mineral oxygenation. Environ. Sci. Technol..

[40] Page S. E., Sander M., Arnold W. A., McNeill K. (2012). Hydroxyl radical formation
upon oxidation of reduced humic acids by oxygen in the dark. Environ. Sci. Technol..

[41] Francioso A., Conrado A. B., Mosca L., Fontana M. (2020). Chemistry and biochemistry
of sulfur natural compounds: Key intermediates of metabolism and redox
biology. Oxid. Med. Cell. Longevity.

[42] Dai H., Wu B., Chen B., Ma B., Chu C. (2022). Diel fluctuation of
extracellular reactive oxygen species production in the rhizosphere
of rice. Environ. Sci. Technol..

[43] Liu J., Shen S., Zhu K., Li Z., Chen N., Lichtfouse E., Jia H. (2024). Novel insights into
the factors influencing
rhizosphere reactive oxygen species production and their role in polycyclic
aromatic hydrocarbons transformation. Soil Biol.
Biochem..

[44] Hanna E., Keller J. K., Chang D., de Bruyn W., Zalman C. (2020). The potential
importance of methylated substrates in methane production within three
northern Minnesota peatlands. Soil Biol. Biochem..

[45] Hawthorne S. E. G., Tsola S. L., Carrión O., Todd J. D., Eyice Ö. (2025). Active
microorganisms and potential metabolic pathways mediating anaerobic
degradation of DMSP in anoxic saltmarsh sediment. ISME Commun..

[46] Tebbe D. A., Gruender C., Dlugosch L., Lõhmus K., Rolfes S., Könneke M., Chen Y., Engelen B., Schäfer H. (2023). Microbial drivers of DMSO reduction and DMS-dependent
methanogenesis in saltmarsh sediments. ISME
J..

[47] Zhuang G.-C., Lin Y.-S., Bowles M. W., Heuer V. B., Lever M. A., Elvert M., Hinrichs K.-U. (2017). Distribution and isotopic composition
of trimethylamine, dimethylsulfide and dimethylsulfoniopropionate
in marine sediments. Mar. Chem..

[48] Carrión O., Curson A. R. J., Kumaresan D., Fu Y., Lang A. S., Mercadé E., Todd J. D. (2015). A novel pathway
producing dimethylsulphide
in bacteria is widespread in soil environments. Nat. Commun..

[49] Carrión O., Pratscher J., Curson A. R. J., Williams B. T., Rostant W. G., Murrell J. C., Todd J. D. (2017). Methanethiol-dependent dimethylsulfide
production in soil environments. ISME J..

[50] Zhou X., Mopper K. (1990). Determination of photochemically
produced hydroxyl
radicals in seawater and freshwater. Mar. Chem..

[51] Yang K., Liu J., Wang Z., Zhu K., Jia B., Yang H., Qin J., Xie J., Latif J., Liu F., Li Y., Chen N., Jia H. (2025). Spatiotemporal dynamics of reactive
oxygen species in the detritusphere and their critical roles in organic
carbon mineralisation. Soil Biol. Biochem..

[52] Barcellos D., Pérez Castro S., Campbell A., Kimbrel J. A., Blazewicz S. J., Wollard J., Pett-Ridge J., Thompson A. (2025). Duration of O_2_ exposure determines dominance of Fe­(II) vs CH_4_ production
in tropical forest soils. Environ. Sci. Technol..

[53] Fan K., Lin C., Li L., Huang Q., Dai J., Wang P., Qin J., Lim J. W., Qiu R. (2015). Rainwater-derived reactive oxygen
species diminish environmental risk from arsenic in paddy rice systems. Environ. Sci. Technol..

[54] Chen N., Fu Q., Wu T., Cui P., Fang G., Liu C., Chen C., Liu G., Wang W., Wang D., Wang P., Zhou D. (2021). Active iron phases regulate the abiotic
transformation of organic carbon during redox fluctuation cycles of
paddy soil. Environ. Sci. Technol..

[55] Neue H. U., Wassmann R., Kludze H. K., Bujun W., Lantin R. S. (1997). Factors
and processes controlling CH_4_ emissions from rice fields. Nutr. Cycling Agroecosyst..

[56] Yang T., He Q., Jiang J., Sheng L., Jiang H., He C. (2022). Impact of
water table on CH_4_ emission dynamics in terrestrial wetlands
and implications on strategies for wetland management and restoration. Wetlands.

[57] Segarra K. E. A., Schubotz F., Samarkin V., Yoshinaga M. Y., Hinrichs K.-U., Joye S. B. (2015). High rates of anaerobic
methane oxidation
in freshwater wetlands reduce potential atmospheric methane emissions. Nat. Commun..

[58] Roslev P., King G. M. (1996). Regulation of methane
oxidation in a freshwater wetland
by water table changes and anoxia. FEMS Microbiol.
Ecol..

