# Supplementary material for: Abiotic Methane Production Driven by Soil Reactive Oxygen Species
Source: Environ Sci Technol. 2026 Apr 6;60(22):15821–9. doi: 10.1021/acs.est.6c01306 (PMC13262033; doi:10.1021/acs.est.6c01306)
Supplement: Supplementary file 1 [file es6c01306_si_001.pdf]

Supporting Information

Abiotic Methane Production Driven by Soil Reactive Oxygen Species

Zi-Yan Liu†,‡, Hao Liu†,⊥, Shi-Yu Zhang†, You Feng†, Ling-Li Wei†, Dongmei Zhou§, and Zheng Chen*,†

†Department of Health and Environmental Sciences, Xi'an Jiaotong-Liverpool University, 111 Ren'ai Road, Suzhou, Jiangsu 215123, P. R. China

‡Department of Chemistry, University of Liverpool, Crown Street, Liverpool, L697ZD, United Kingdom

⊥Centre for Metabolomics Research, Department of Biochemistry, Cell and Systems Biology, Institute of Systems, Molecular and Integrative Biology, University of Liverpool, Liverpool L697BE, United Kingdom

§State Key Laboratory of Pollution Control and Resource Reuse, School of the Environment, Nanjing University, Nanjing 210023, Jiangsu Province, P. R. China

***Corresponding author**:
Zheng Chen: ebiogeochem@outlook.com & Zheng.Chen@xjtlu.edu.cn
Department of Health and Environmental Sciences, Xi'an Jiaotong-Liverpool University, 111 Ren'ai Road, Suzhou 215123, China. Tel: +86-512-81880471; Fax: +86-512-88161899

This material includes 10 pages, including 6 figures and 2 tables.

S1. Supporting Tables........S3

Table S1. Geographic Location and Physicochemical Properties of the 14 Soil Samples Used in This Study. S3

Table S2. Methane and Hydroxyl Radical Production in Sterilized Soil Slurries from 14 Wetland Sites after 8, 24, and 360 hours of Oxygenation with 100 mM DMSO. S4

S2. Supporting Figures S5

Figure S1. CH_4 production under different oxygenation and substrate conditions in sterilized soil. S5

Figure S2. Temporal variation in •OH accumulation during oxygenation for different anaerobic incubation times. S6

Figure S3. Temporal variation of (a) •OH accumulation, (b) the reduction in Fe(II) proportion, and (c) CH_4 concentration during 72 hours of oxygenation. S7

Figure S4. Relationships between anaerobic incubation, Fe(II) reduction, •OH accumulation, and CH_4 production. S8

Figure S5. Schematic workflow of the soil microcosm experiments. S9

Figure S6. Photograph of the experimental setup for the drainage simulation. S10

S1. Supporting Tables

Table S1. Geographic Location and Physicochemical Properties of the 14 Soil Samples Used in This Study.

Site Name	Abbr.	Province	Lat. (°N)	Long. (°E)	Soil Type	pH	TOC (g/kg)	TN (g/kg)	Amorph. Fe (g/kg)
Baisha	BS	Hainan	19.23	109.45	Latosol	5.4	12.3	1.1	15.6
Yuedong	YD	Guangdong	23.06	113.26	Lat. Red Earth	5.8	15.7	1.3	18.2
Baoding	BD	Hebei	38.85	115.48	Cinnamon	7.5	18.9	1.5	11.5
Panjin	PJ	Liaoning	41.12	122.07	Saline	8.2	22.4	1.8	9.8
Wuchang	WC	Heilongjiang	44.92	127.15	Black	6.5	35.1	2.5	12.1
Chenzhou	CZ	Hunan	25.8	113.03	Red Earth	5.2	14.5	1.2	20.4
Suzhou	SZ	Jiangsu	31.3	120.62	Paddy	6.8	20.1	1.7	14.3
Ganzhou	GZ	Jiangxi	25.83	114.93	Red Earth	5.5	13.8	1.1	22.1
Yantai	YT	Shandong	37.53	121.4	Brown	7.1	16.2	1.4	13.5
Aba	AB	Sichuan	31.9	102.22	Alpine Meadow	6.2	45.3	3.5	8.7
Mianyang	MY	Sichuan	31.47	104.75	Purple	7.8	19.5	1.6	10.9
Xinjiang	XJ	Xinjiang	43.82	87.62	Gray Desert	8.5	8.9	0.8	7.5
Kunming	KM	Yunnan	25.04	102.71	Red Earth	6.1	25.6	2.1	19.8
Zhoushan	ZS	Zhejiang	30.01	122.21	Coastal Saline	7.9	21.8	1.9	16.7

Table S2. Methane and Hydroxyl Radical Production in Sterilized Soil Slurries from 14 Wetland Sites after 8, 24, and 360 hours of Oxygenation with 100 mM DMSO. (Values are mean ± standard deviation, n=3).

**Site (Abbr.)**	**CH_4 ($\mu mol \cdot L^{-1}$) 8h**	**•OH ($nmol \cdot L^{-1}$) 8h**	**CH_4 ($\mu mol \cdot L^{-1}$) 24h**	**•OH ($nmol \cdot L^{-1}$) 24h**	**CH_4 ($\mu mol \cdot L^{-1}$) 360h**	**•OH ($nmol \cdot L^{-1}$) 360h**
BS	0.95 ± 0.11	25.4 ± 2.1	1.80 ± 0.19	45.6 ± 4.2	3.47 ± 0.37	88.3 ± 9.1
YD	0.50 ± 0.06	15.2 ± 1.5	0.77 ± 0.09	22.8 ± 2.4	1.12 ± 0.12	35.1 ± 3.7
BD	1.28 ± 0.14	30.1 ± 3.3	2.23 ± 0.24	50.2 ± 5.5	4.26 ± 0.45	95.6 ± 10.1
PJ	1.76 ± 0.19	40.5 ± 4.1	3.44 ± 0.36	75.3 ± 7.9	7.56 ± 0.80	145.2 ± 15.3
WC	1.38 ± 0.16	33.6 ± 3.6	2.54 ± 0.27	58.9 ± 6.2	5.13 ± 0.54	110.4 ± 11.6
CZ	1.60 ± 0.17	38.2 ± 3.9	3.05 ± 0.32	68.7 ± 7.2	6.15 ± 0.65	125.8 ± 13.2
SZ	2.50 ± 0.26	55.3 ± 5.8	4.88 ± 0.51	98.4 ± 10.3	9.69 ± 1.02	180.1 ± 18.9
GZ	2.23 ± 0.23	50.1 ± 5.3	4.26 ± 0.44	88.2 ± 9.2	8.15 ± 0.85	155.9 ± 16.4
YT	1.18 ± 0.13	28.4 ± 3.0	2.04 ± 0.22	48.1 ± 5.1	3.75 ± 0.39	85.3 ± 9.0
AB	2.82 ± 0.30	60.7 ± 6.4	5.51 ± 0.58	110.2 ± 11.6	10.96 ± 1.15	201.3 ± 21.1
MY	3.14 ± 0.33	68.9 ± 7.2	6.22 ± 0.65	125.4 ± 13.2	12.19 ± 1.28	220.7 ± 23.2
XJ	1.54 ± 0.16	35.9 ± 3.8	2.84 ± 0.30	65.1 ± 6.8	5.63 ± 0.59	118.2 ± 12.4
KM	1.88 ± 0.20	42.8 ± 4.5	3.67 ± 0.39	80.3 ± 8.4	7.21 ± 0.75	140.5 ± 14.8
ZS	2.41 ± 0.26	52.1 ± 5.5	4.70 ± 0.49	95.6 ± 10.0	9.28 ± 0.97	175.2 ± 18.4

S2. Supporting Figures

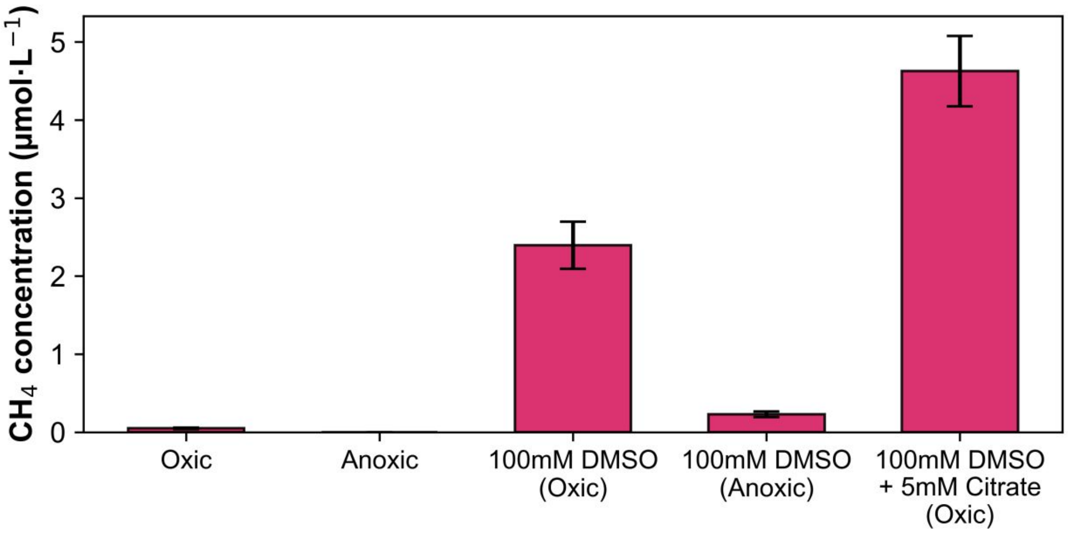

Figure S1. CH_4 production under different oxygenation and substrate conditions in sterilized soil. Treatments involved sterilized soil under oxic conditions (Oxic); anaerobic conditions (Anoxic); with 100 mM DMSO under oxic conditions; with 100 mM DMSO under anaerobic conditions; and with 100 mM DMSO and 5 mM citric acid under oxic conditions. Methane levels varied significantly, indicating the influence of oxygen and substrate availability.

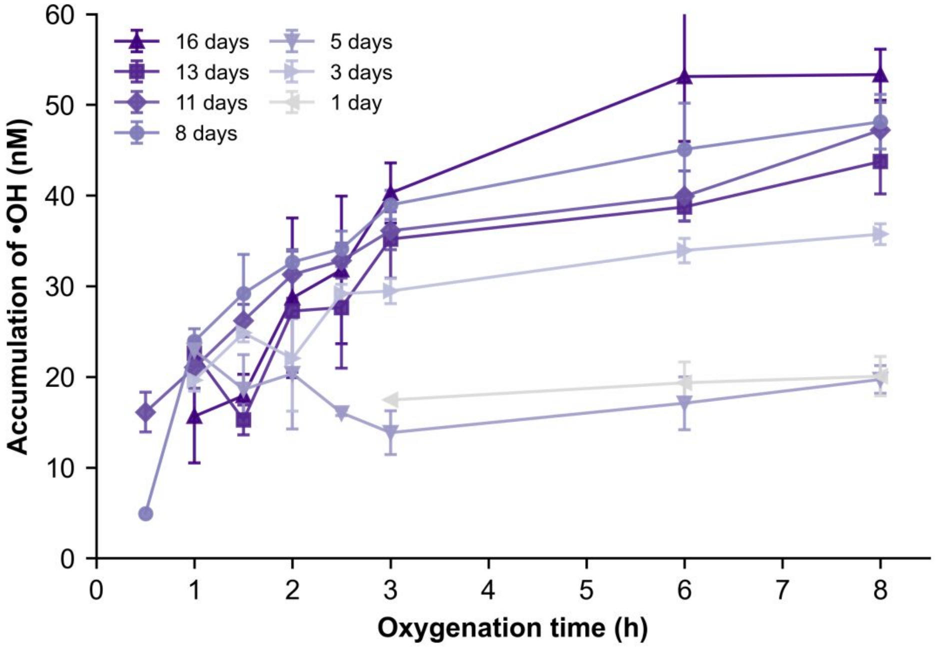

Figure S2. Temporal variation in •OH accumulation during oxygenation for different anaerobic incubation times. Samples were incubated anaerobically for 1, 3, 5, 8, 11, 13, or 16 days prior to oxygenation. Longer anaerobic incubation led to higher •OH accumulation upon subsequent oxygenation.

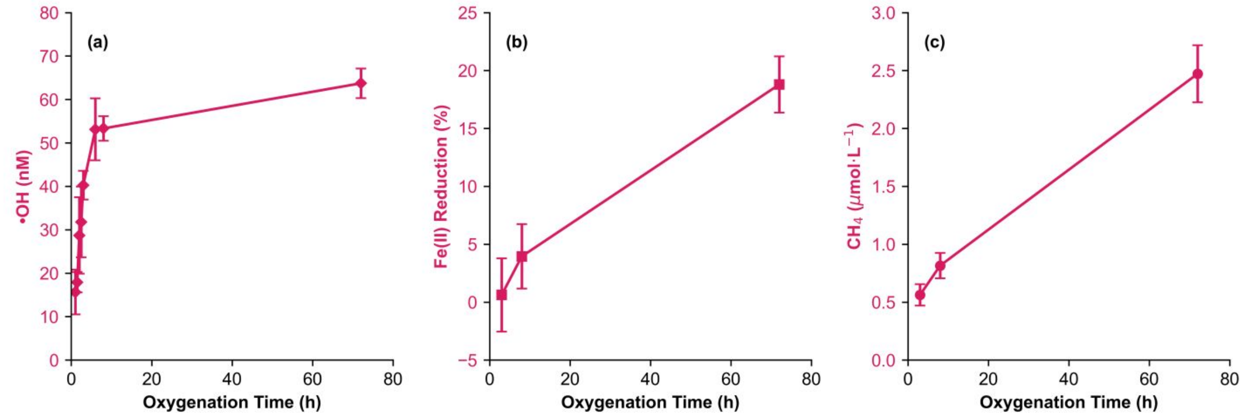

Figure S3. Temporal variation of (a) • OH accumulation, (b) the reduction in Fe(II) proportion, and (c) CH_4 concentration during 72 hours of oxygenation. The results are from Zhoushan soil slurry that was previously incubated anaerobically for 16 days. Error bars represent standard deviation (n=3).

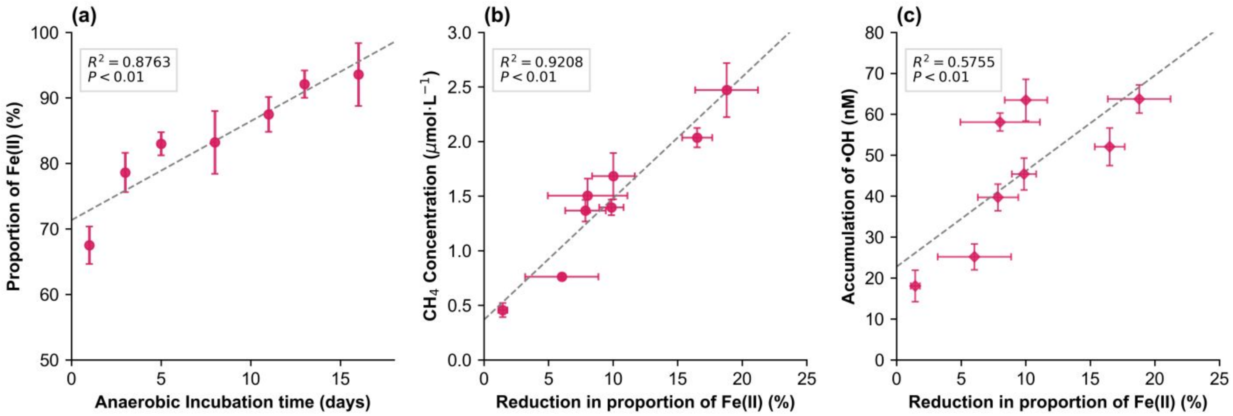

Figure S4. Relationships between anaerobic incubation, Fe(II) reduction, •OH accumulation, and CH_4 production. in anaerobic Zhoushan soil slurry after 72 hours of oxygenation. **(a)** Fe(II) proportion as a function of anoxic incubation time. **(b)** CH_4 yield as a function of Fe(II) reduction. **(c)** •OH accumulation as a function of Fe(II) reduction.

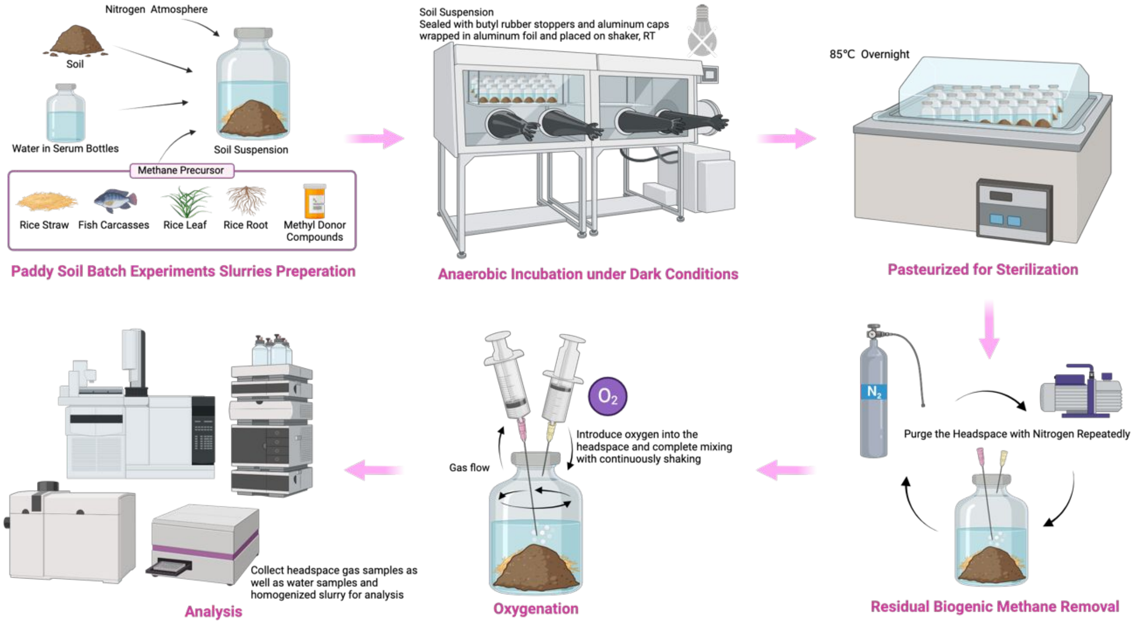

Figure S5. Schematic workflow of the soil microcosm experiments. This diagram illustrates the key steps from sample preparation and anaerobic incubation to oxygenation and analysis.

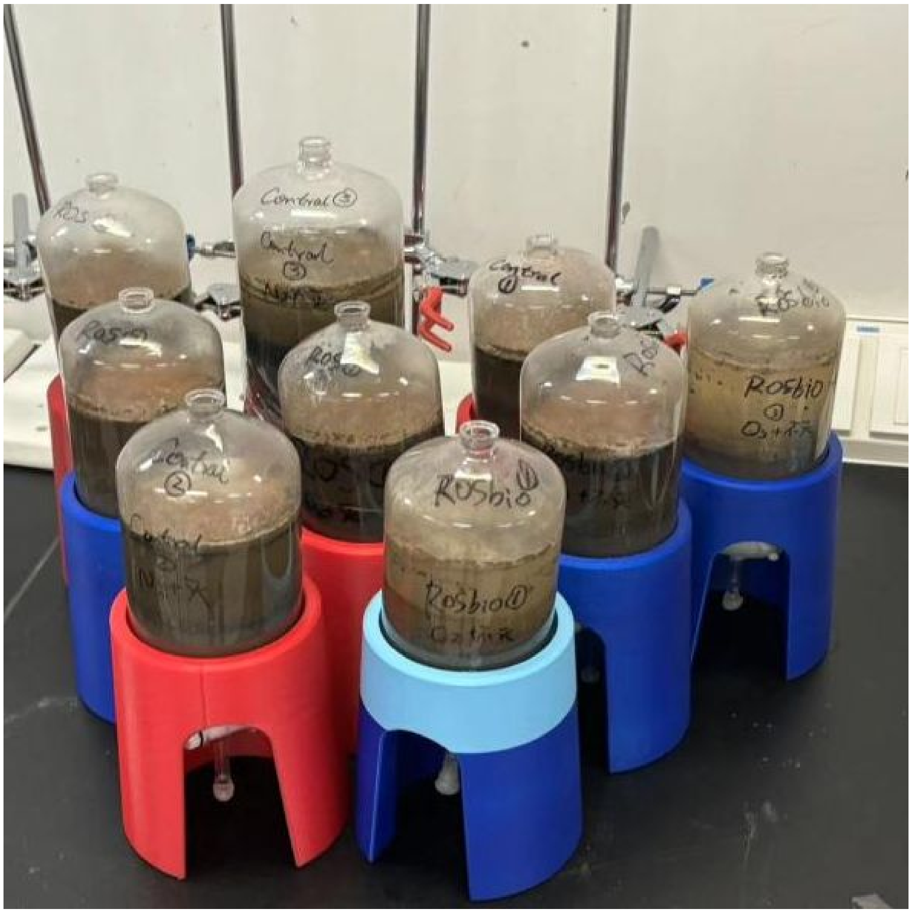

Figure S6. Photograph of the experimental setup for the drainage simulation. The image shows the soil columns used to replicate field-like drainage conditions.
